# A new paradigm for diagnosis of neurodegenerative diseases: peripheral exosomes of brain origin

**DOI:** 10.1186/s40035-022-00301-5

**Published:** 2022-05-09

**Authors:** Neelam Younas, Leticia Camila Fernandez Flores, Franziska Hopfner, Günter U. Höglinger, Inga Zerr

**Affiliations:** 1 Prion Research Group, Department of Neurology, National Reference Center for Surveillance of TSE, Universitätsklinikum Göttingen: Universitätsmedizin Göttingen, Georg-August University, Göttingen, Germany, Robert-Koch-Strasse 40, 37075 Göttingen, Germany; 2 Hannover Medical School, Medizinische Hochschule Hannover, Hannover, Germany; 3 German Center for Neurodegenerative Diseases (DZNE), Munich, Germany; 4 German Center for Neurodegenerative Diseases (DZNE), Göttingen, Germany

**Keywords:** Alzheimer’s disease, Central nervous system, Diagnosis, Exosomes, Blood–brain barrier, Parkinson’s disease

## Abstract

Neurodegenerative diseases are a heterogeneous group of maladies, characterized by progressive loss of neurons. These diseases involve an intricate pattern of cross-talk between different types of cells to maintain specific signaling pathways. A component of such intercellular cross-talk is the exchange of various types of extracellular vesicles (EVs). Exosomes are a subset of EVs, which are increasingly being known for the role they play in the pathogenesis and progression of neurodegenerative diseases, e.g., synucleinopathies and tauopathies. The ability of the central nervous system exosomes to cross the blood–brain barrier into blood has generated enthusiasm in their study as potential biomarkers. However, the lack of standardized, efficient, and ultra-sensitive methods for the isolation and detection of brain-derived exosomes has hampered the development of effective biomarkers. Exosomes mirror heterogeneous biological changes that occur during the progression of these incurable illnesses, potentially offering a more comprehensive outlook of neurodegenerative disease diagnosis, progression and treatment. In this review, we aim to discuss the challenges and opportunities of peripheral biofluid-based brain-exosomes in the diagnosis and biomarker discovery of Alzheimer’s and Parkinson’s diseases. In the later part, we discuss the traditional and emerging methods used for the isolation of exosomes and compare their advantages and disadvantages in clinical settings.

## Background

A common feature of neurodegenerative diseases is the misfolding, aggregation and accumulation of pathological amyloids inside or outside of the brain cells [1]. Accordingly, the detection of these pathological proteins in body fluids and accessible tissues may be an ideal candidate for early diagnosis of these diseases [2, 3]. However, due to the significant differences in their concentrations among various tissues and biofluids, the detection of these protein aggregates, in most cases by standard assays, is only possible in the affected brain [3]. A great progress has been made in the development of central nervous system (CNS)-proximal biomarkers, including PET-neuroimaging and cerebrospinal fluid (CSF) measures of tau, beta-amyloid, α-synuclein and prion protein. CSF biomarkers are useful as screening tools and as supplementary information to diagnostic examinations, but cannot be used as diagnostic tools in isolation [4]. The changes in the CSF levels of pathological proteins reflect the ongoing deposition of these proteins in the brain [5–7]. Screening of biomarkers in the CSF through a lumbar puncture is invasive, complex and expensive. In addition, frequent sampling at several intervals is not easy to achieve.

Although a great progress has been made in CSF-based biomarkers for the diagnosis of neurodegenerative diseases, there are also important unmet needs. A major caveat in the development of peripheral biomarkers is the extremely low concentration of pathological proteins, i.e., less than one ten-billionth of total blood protein and one millionth of total CSF protein [8]. Therefore, highly sensitive methods are required for specific detection of pathological protein aggregates. The real-time quaking induced conversion (RT-QuIC) assay has emerged as a robust, fast and ultrasensitive tool for template-mediated amplification of misfolded protein aggregates in the CSF [3, 9]. However, due to the inhibitory factors in the blood, which disturb the seeding amplification of aggregated proteins, a blood-based RT-QuIC assay remains a challenge. One strategy to develop sensitive and effective biomarkers is to concentrate on the constituents of body fluids other than soluble proteins [10], e.g. extracellular vesicles. Identification of peripheral biomarkers could enable sensitive antemortem diagnosis of Parkinson’s disease (PD), Alzheimer’s disease (AD) and other neurodegenerative disorders.

A growing body of literature has highlighted an important role of EVs in the cell-to-cell transmission of pathogenic protein aggregates, thereby contributing to the pathological and clinical progression of neurodegenerative diseases [11, 12].

Increasing evidence has shown that small EVs (for example exosomes) can function as biomarkers for neurodegenerative diseases and that they are more reliable than conventional specimens, such as pure CSF, blood and urine [13]. Finally, they can cross the blood–brain barrier [14] and have low immunogenicity [15], thereby receiving much attention as potential biomarkers and drug delivery tools for the treatment of neurodegenerative diseases. However, there are several challenges in implementing exosomes as biomarkers, such as inefficient separation methods, lack of high-resolution visualization techniques and lack of standardization in establishing high-throughput methods. Another challenge is the isolation of low-abundance exosomes released from the brain into the body fluids, e.g. blood [12]. Therefore, ultrasensitive and highly efficient methods are required to obtain substantial breakthroughs.

The biomarker potential of total EVs and associated pitfalls have been discussed heavily in the literature. However, the emerging potential of brain-derived cell-specific exosomes and associated caveats need to be discussed to bring them into clinics. In this review, we aim to discuss the emerging biomarker potential of CNS-exosomes isolated from peripheral bio-fluids for early diagnosis of neurodegenerative diseases, including AD, synucleinopathies and prion diseases. Particularly, we want to bridge the gap to bring exosome technology into the clinics. For this, we also discuss the advantages and disadvantages of traditional and latest methods used to isolate exosomes; examples are the relative extent of development of a method, purity, time, scalability, throughput and cost, all of which are important aspects for bringing exosome technology into clinical applications.

## Extracellular vesicles

EVs are a heterogeneous group of cell-derived membranous vesicles, which are secreted into the extracellular space [16]. EVs are classified into three main subtypes based on size and the nature of biogenesis: exosomes, microvesicles (MVs) and apoptotic bodies [17]. Apoptotic bodies are the largest EVs, measuring ∼500–4000 nm, which are formed as a result of programmed cell death [18]. MVs, also known as microparticles, ectosomes or shedding vesicles, have a characteristic size of ∼100–1000 nm. MVs are formed by direct outward budding of the plasma membrane [19]. MVs are distinguished from apoptotic bodies by size, their biogenesis, cargo and membrane-specific markers, as they originate from the plasma membrane [20].

Exosomes are defined specifically by their diameter (∼30–150 nm) [21] and exosomal markers, e.g., Alix, TSG101, HSC70, HSP90β, and tetraspanins (CD81, CD9, CD63) [22]. Exosomes are generated from late endosomes. Inward budding of late endosomes captures cytoplasmic biomolecules, and produces intraluminal vesicles (exosomes) within multivesicular bodies (MVBs). The MVBs fuse with the plasma membrane, releasing exosomes into the extracellular space [23] where they can be taken up by target cells [24]. Since the discovery of exosomes in the late 1980s, they were initially considered as mere cellular waste bins [25]. However, with the advancement in research technologies, it has been discovered that exosomes represent a new mode of intercellular communication and contribute to a wide range of physiological and pathological processes [26]. Exosomes are released by virtually all cell types, including neurons [27], oligodendrocytes [28], microglia [29] and astrocytes [30].

Exosomes contain bioactive cargos, such as lipids, metabolites, proteins and nucleic acids [31], which can be transferred to the target cells. According to the EXOCARTA database (www.exocarta.org), 41,860 proteins, 2838 miRNAs, 3408 mRNAs, and 1116 lipids have been identified from exosomes of different cell types [32]. Exosomes are present in various biofluids, such as cerebrospinal fluid [33], blood [34], urine [35] and saliva [36] among others. These lipid-bilayer vesicles are very stable and can protect their molecular contents from degradation and denaturation in the extracellular environments [37].

The nomenclature used for EVs is somewhat problematic. The International Society for Extracellular Vesicles consistently assists in the definitions and isolation methods of EV; however, inconsistent usage remains a problem [38, 39]. Many studies have used the terms exosomes and EVs interchangeably in the literature. Here, we use the term “exosomes” exclusively when describing the studies that go through the characterization of EV preparations enriched with exosomes. It is worth noting that in many studies, the exosomes prepared might also contain small amounts of other EVs.

A lot of focus has been given to exosomes in neurodegenerative diseases, e.g., neuronal, astrocytic and oligodendrocytic exosomes [40–43], while the utility of other EV subtypes as biomarkers of neurodegenerative diseases has not yet been fully elucidated and requires further attention. Little evidence is available in the literature for MV alterations in neurodegeneration [44]. One plausible reason for this could be that most investigations do not clearly differentiate between exosomes, MVs and apoptotic bodies [44]. A very recent study showed changes in neuronal MVs isolated from CSF [45]. However, there is still a lack of information whether neuronal MVs can also be detected in blood just like exosomes [45, 46]. In this regard, the main hurdle is the lack of specific markers and technical approaches to isolate pure populations of MVs [47]. Although exosome research also faces similar challenges, there is a growing body of investigations on the role of exosomes and their biomarker potential for neurodegenerative diseases, in comparison to other subtypes.

*Why are exosomes ideal nanoparticles as diagnostic tools in neurodegenerative diseases?*

Exosomes have great potential as diagnostic tools and biomarkers for neurodegenerative diseases due to the following three reasons [48].

1. Exosomal cargos (proteins, various RNA species) are altered during neurodegenerative diseases [13, 48],
2. Exosomes can cross the blood–brain barrier in a bi-directional manner [49–51],
3. Availability of surface markers to capture exosomes of the CNS origin, which can be potentially used to identify their cellular origin [12].

There has been a great interest on the peripheral exosomes of brain origin [12, 52]. These exosomes could not only provide information for the understanding of pathogenesis of neurodegenerative diseases [53, 54], but also allow measuring the extent of neurodegeneration in real time [55]. This would offer a great solution as there are no real efficient and sensitive biomarkers to detect the earliest stages of neurodegenerative diseases (AD and PD), which are affecting an increasing number of people worldwide [56].

### Biomarkers from CSF-isolated CNS-exosomes

**Fig. 1:**
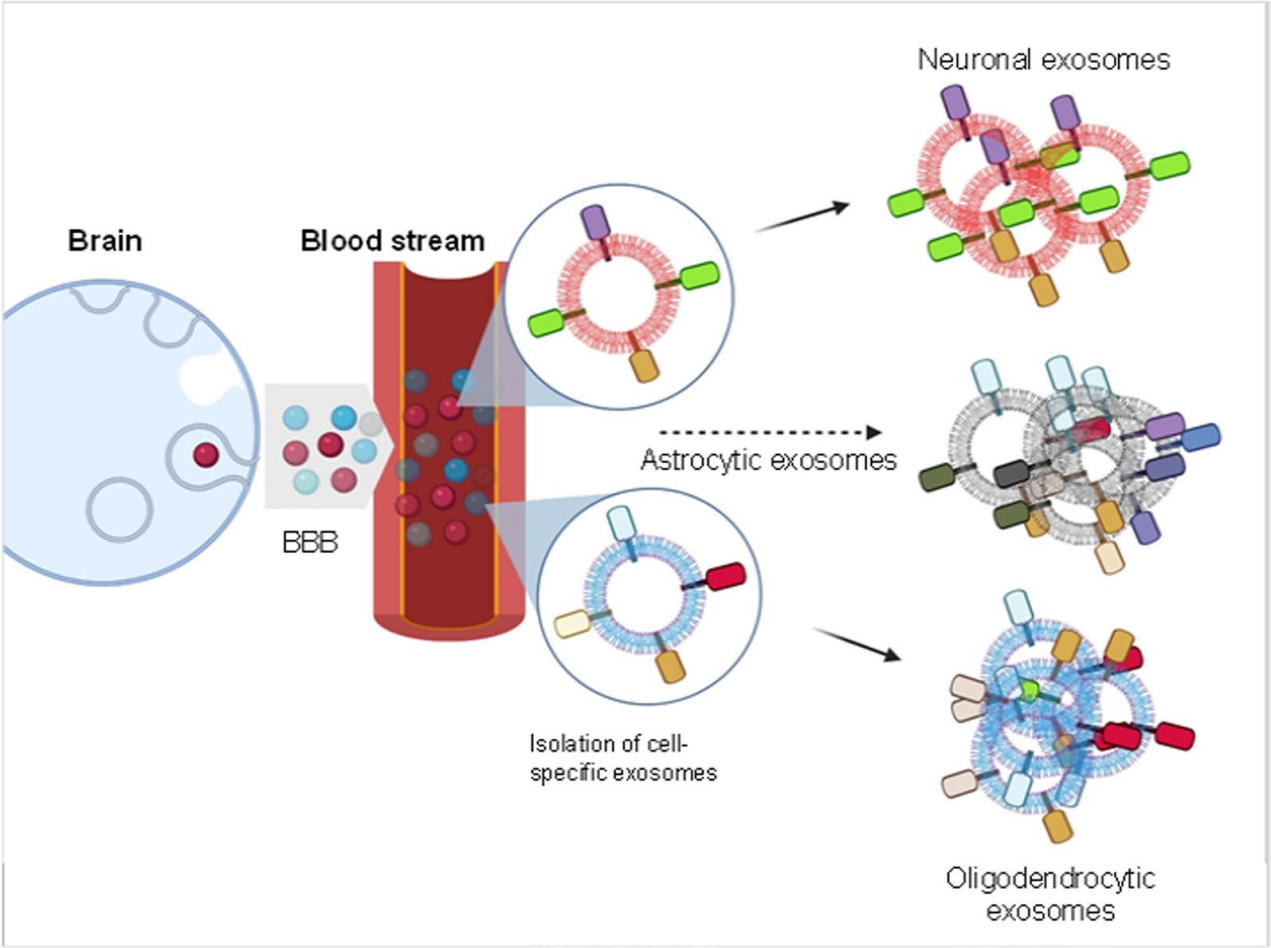
Different types of brain-originated exosomes (neuronal, astrocytic, and oligodendrocytic origin) can cross the blood–brain barrier (BBB) into the bloodstream. These exosomes can be isolated from blood using surface markers specific to parental cells (Created in Biorender.com)

The enrichment of brain cell-specific exosomes, such as neuronal, astrocytic, and more recently, oligodendrocytic exosomes, has opened up a new paradigm for the diagnosis of neurodegenerative diseases. Specific surface markers allow the enrichment of CNS-cell specific exosomes. For example, neural cell adhesion molecule L1 (L1CAM), neural cell adhesion molecule (NCAM), and GluR2/3 (GluR2/3 subunits of α-amino-3-hydroxy-5-methyl-4-isoxazole propionic acid) are used for the isolation of neuronal exosomes [12, 14, 57, 58], glutamine aspartate transporter (GLAST) is used for the isolation of astrocytic exosomes [59], while Myelin proteolipid protein [14] and myelin oligodendrocyte glycoprotein (MOG) [60] are used for the isolation of oligodendrocyte-specific exosomes. Due to the absence of a unique microglial surface marker, isolation of microglial exosomes is still an unmet challenge (Fig. 1). Although immunocapturing using these surface markers allows the enrichment of cell-specific exosomes, there are several challenges associated with the extraction, which will be discussed later.

CSF is one of the main biological fluids used for the detection of exosomes, particularly neuronal exosomes. The levels of phosphorylated tau (p-tau, T181) in CSF-isolated exosomes increase at early stages (Braak stage 3) and decrease at later stages (Braak stage 5–6) [61]. Although this is a promising finding, it requires validation in appropriate cohorts. The exosome preparations used in this study were isolated by ultracentrifugation followed by sucrose fractionation. The results indicate that the exosome-mediated secretion of p-tau may lead to abnormal processing of tau, and the genesis of elevated levels of CSF p-tau at early stages of AD [61]. Later investigations also using the ultracentrifugation method for isolation of EVs have shown the presence of other prion/prion-like proteins (e.g. tau and prion protein) in the CSF-isolated EVs [62]. Furthermore, full-length tau protein has been identified in CSF-derived exosomes from healthy individuals [63]. The early detection of exosomal p-tau indicates a biomarker potential of exosomal tau for preclinical diagnosis of AD.

Pathogenic form of α-synuclein has also been detected in the CSF-isolated exosomes from PD patients at early stages and patients of dementia with Lewy bodies, which has shed light on several aspects of α-synuclein spreading and clearance [64]. Under the background that previous efforts measuring α-synuclein concentration in the CSF did not successfully differentiate PD patients from healthy subjects [65], Stuendl et al. highlighted the biomarker potential of EV-bound α-synuclein [64, 66]. Another study reported a Class III evidence (NPub.org/coe) that the CSF EVs positive for total and aggregated α-synuclein can identify patients with PD. The measurement was performed using antibodies against total or aggregated α-synuclein. The results showed a lower amount of total or aggregated α-synuclein in PD patients in comparison with healthy controls [67]. Although the findings are promising, the potential of CSF exosomal α-synuclein as a diagnostic marker needs further investigations [64]. However, due to its close connection with the brain, CSF-EVs can be used as a reference for peripheral exosomes analysis. In addition to the misfolded proteins, exosomal miRNAs and RNAs have also garnered great interest in either detecting or perpetuating neurodegenerative disease progression. Beyond the scope of this review, the biomarker potential of exosomal miRNAs has been discussed in reviews [68, 69].

Overall, the detection of pathogenic proteins (tau, prion protein and α-synuclein) in CSF-isolated EVs/exosomes, particularly detection of α-synuclein, at earlier stages suggests a great potential of EVs/exosomes to serve as early biomarkers for neurodegenerative diseases. Furthermore, a correlation between brain EV-markers and peripheral biomarkers will be advantageous.

### Blood-derived CNS-exosomal markers

Typically, exosomes are collected from human CSF for the detection of protein markers related to neurodegenerative diseases. However, due to the complicated and painful nature of CSF collection, isolation of exosomes from CSF is not very feasible. Therefore, human blood-mediated detection will be a simple and powerful approach, since brain-secreted exosomes can cross the blood–brain barrier, and be collected from blood (serum and plasma) [12] (Fig. 1). Immunoisolation can be used to purify brain-derived exosomes from blood samples by targeting surface markers. To isolate neuronal exosomes from blood, most of the investigations have used a commercial exosome precipitation kit named "ExoQuick" followed by immunoprecipitation with anti-human CD171 (also known as L1CAM, a putative CNS-specific marker) [46, 70, 71]. A single investigation has used another neuronal marker NCAM instead of L1CAM [72]. On the contrary, a very recent study has suggested that L1CAM, present in the human plasma, is not EV-associated but is present in soluble fraction, producing doubts about the utility of L1CAM for the isolation of exosomes of neuronal origin [73]. To overcome the limitations associated with current markers, there is an urgent need to develop better markers. In many studies, the isolated exosome preparations might also contain small amounts of other EVs, as the surface markers used to isolate cell-specific exosomes are abundant in respective cell-type but are not exclusive to them. To measure the levels of markers in the isolated exosome preparations, most of the studies have used ELISA-based assays.

High levels of total tau, phosphorylated-tau (T181, S396), Aβ1-42 and hemoglobin have been identified in L1CAM-positive neuronal exosomes isolated from the plasma of patients with AD compared with controls [70, 74]. Although the results are intriguing, longitudinal studies comparing the neuronal-exosomal profiles of cognitively normal individuals having an abnormal neuronal-exosomal profile to those having a normal profile are required to strengthen the clinical usefulness of this neuronal exosome profile.

Several other reports have shown abnormal levels of neuropathogenic proteins and some other markers in L1CAM-positive neuronal exosomes. The levels of transcription factors, including heat-shock factor-1 and repressor element 1-silencing transcription factor, are significantly decreased in AD patients than in controls [70]. Abnormal levels of p-tau, Aβ1-42, neurogranin and the repressor element 1-silencing transcription factor in neuronal exosomes can predict the conversion of mild cognitive impairment to AD. Furthermore, plasma-derived neuronal exosomes from AD patients can seed tau aggregation, leading to AD-like pathology in normal mouse brain [75]. A significant reduction in synaptic proteins, including synaptotagmins, synaptophysin, synaptopodin, synaptobrevin, neurogranin, Rab3A, and GAP, has also been identified in the plasma-derived neuronal exosomes of AD patients [76]. The simultaneous quantification of synaptic proteins from different types of exosomes (e.g. neuronal, astrocytic and oligodendrocytic) in a stage-dependent manner might provide clues about the molecular changes that occur with the progression of the disease.

Recently, a multi-center study has confirmed the viability of NCAM-positive neuron-derived exosomes as a diagnostic tool. The study has reported that exosomal Aβ42, total-tau, and p-tau (T181) have the same ability as those in pure CSF for the diagnosis of mild cognitive impairment and AD [72]. Dysregulation of the insulin pathway proteins in L1CAM-neuron-derived exosomes was identified in AD pateints, allowing a prediction of pre-clinical biomarkers (~ 10 years) before the onset of symptoms [77]. Although the neuronal-exosomal proteins can distinguish between mild cognitive impairment and AD with more accuracy than the free proteins in blood, the isolation process of cell-specific exosomes is quite complex and requires standardization. A panel of different markers from these exosomes may provide reliable prognosis, disease staging, and diagnosis.

The astrocytic exosomal cargo from the plasma of AD patients has also been explored. In a previous study, exosomes were isolated from plasma samples using ExoQuick exosome isolation kit, and astrocytic exosomes were enriched by immunoprecipitation with surface marker GLAST. ELISA quantitation showed that the levels of BACE-1, γ-secretase, sAPPβ, sAPPα, and glial-derived neurotrophic factor  were up to 20-fold higher in comparison to neuron-derived exosomes. In addition, BACE-1 levels were significantly higher in AD patients than controls, while Septin-8 levels were lower in AD patients [59]. At the preclinical stage, 6–11 years before the onset of AD, neuronal-exosomal levels of synaptic proteins including neurexin 2α, GluA4-containing glutamate receptor, and neuroligin 1 were significantly reduced in AD compared to age-matched controls [78]. Further research into these proteins may be beneficial for the detection of preclinical biomarkers and disease severity. Furthermore, side-by-side measurement of several markers from different cell types (e.g. neuronal and astrocytic exosomes) in the same samples may not only provide a robust biomarker panel but also provide new mechanistic insights into the pathogenic processes of the disease.

Neuronal exosomes from the blood samples of PD patients have also been explored. The level of α-synuclein in L1CAM-positive neuronal exosomes in early-stage PD patients was significantly higher than that of healthy individuals [79]. The higher levels of exosomal α‐synuclein were linked to the increased risk of motor progression after a follow-up of 22 months. In addition, PD patients were found to have higher concentrations of tau in plasma-derived L1CAM-positive exosomes, compared to AD patients [80]. Alterations in the total and neuronal exosomal α-synuclein concentrations from plasma have been linked to PD progression [81]. More recently, the measurement of α-synuclein and clusterin levels in L1CAM-positive EVs has been demonstrated as a robust method to differentiate PD from related movement disorders [57, 82]. Oligomeric α-synuclein and some proteins of the SNARE complex, isolated from the L1CAM-positive neuronal exosomes, have also shown biomarker potential for the diagnosis of PD [83].

Similarly, Dutta et al. isolated neuronal and oligodendroglial exosomes from plasma/serum of multiple system atrophy (MSA) or PD patients by immunoprecipitation using L1CAM or anti-MOG. They found that the ratio of α-synuclein in oligodendroglial to neuronal exosomes could distinguish between PD and MSA patients with high sensitivity and specificity [60].

Another study indicated that tau and Aβ1-42 in plasma EVs are significant markers of cognitive function in PD patients based on the artificial neural network models. This investigation highlights the prognostic roles of plasma-EV tau and Aβ1-42 in PD. EVs in this study were isolated using the exoEasy Maxi kit, and confirmed to be mainly exosomes by the presence of exosomal markers CD9, CD63 and TSG 101. The levels of tau and Aβ1-42 in the EVs were measured by immunomagnetic reduction assay [84].

Several studies have documented the role of exosomes in the pathogenesis of prion diseases [85–90]. However, the biomarker potential of exosomal-derived prion protein remains unexplored.

In summary, these findings highlight the potential of blood-derived CNS-exosomes as a source of diagnostic, prognostic, and progression biomarkers for neurodegenerative disorders. An added advantage of biomarker examination in blood-derived brain exosomes is the capability to compare these biomarkers originating from different cell types, side-by-side in the same sample, due to the routine availability and much less invasive nature of blood sampling [42].

Although the blood-isolated CNS exosomes offer a great opportunity to study neurodegenerative diseases outside of the brain and CSF, there are several concerns about the “black box” nature of isolation of cell-specific exosomes. Little is known about how exosome production from different cell types is influenced by different disease states and stages. The observed differential expression of exosomal proteins could be due to an increase in the number of exosomes or in the concentration of a specific protein. Can a change in the cell surface proteins used to isolate the exosomes affect the measured concentrations, particularly if this change is a result of a biological process? Further research on exosome biology, isolation and detection methods will help answer these questions and advance the field of exosome-based diagnostics.

### Saliva-based CNS exosomal markers

Saliva is an easily accessible biofluid composed of serous and mucous secretions [91]. Salivary exosome isolation is a non-invasive, painless, and relatively simple procedure in comparison to blood sampling [92]. Recently, the biomarker potential of salivary exosomes has been explored in PD. In the salivary exosomes isolated with XYCQ EV enrichment kit, the level of absolute α-synuclein oligomers and the ratio of α-synuclein oligomers/total α-synuclein are higher in PD patients compared to the control subjects [93, 94]. Another study has shown that the levels of neuronal exosomes in saliva isolated with polyethylene glycol precipitation increase in PD patients compared to the healthy controls [94]. Biomarkers based on non-invasively obtained exosomes such as those from saliva would be very suitable for clinical applications. Until recently, very limited investigations have been performed on the utility of salivary neuronal exosomes. In conclusion, the significant results obtained from PD patients suggest that the biomarker potential of salivary neuronal exosomes should be expanded in other neurodegenerative diseases as well.

### Urine-based exosomal markers

The low levels of exosomes in biological fluids present detection challenges. The heterogeneity of vesicle sources in body fluids, and the variability of turnover and half-life of EVs are still enigmatic in clinical settings. Compared to other body fluids, urine can be obtained non-invasively and in higher amounts, enabling multiple analyses with a range of existing technologies. Until recently, investigations in the urinary exosomal-biomarker field have focused particularly on kidney and metastatic cancers, considering kidneys as a source of urinary EVs. A very recent study has shown that the urine population of EVs is more heterogeneous than previously assumed [95]. The proteome of urine-derived EVs isolated by differential centrifugation contains proteins that delineate organs across the whole body. Most strikingly, enrichment of neurodegenerative disease-linked proteins has been reported in the urinary EV proteome. The levels of SNAP23 and calbindin proteins are elevated in PD patients [95] in comparison to controls. This study also showed that of the EV-identified proteome, most of the proteins linked with neurological diseases are among the proteins with highest stability in levels from week to week (relative standard deviation < 50%) [95].

Another study has reported the detection of PD-related proteins, DJ-1 and LRRK2, in urine-derived exosomes isolated using the microfiltration method, with a significant sex difference for the level of DJ-1 [96]. The level of urinary exosome-isolated phosphorylated LRRK2 (Ser-1292) was shown to be higher in male patients with idiopathic PD compared to the females [97]. Wang et al. [98] also showed the efficacy of phosphorylated LRRK2 in the urinary EVs in the diagnosis of male PD patients. In both studies, the urinary EVs were isolated by differential centrifugation.

Overall, the positive detection of PD-associated biomarker candidates provides evidence that urinary EVs may be an underutilized source for biomarker discovery, particularly for neurodegenerative diseases [95].

Very recently, the utility of urinary exosomes in the diagnosis of early-stage AD has also been documented. The levels of hallmark proteins of AD, Aβ1-42 and tau (P-S396) in urinary exosomes, were elevated in AD patients compared to the healthy subjects. In this study, urinary exosomes were isolated by the ExoQuick Exosome Precipitation kit, followed by transmission electron microscopy and nanoparticle tracking analysis. A difference was found in the abundance and size of exosomes, providing further evidence that the urine-derived exosomes are potential diagnostic markers for AD [99].

**Fig. 2:**
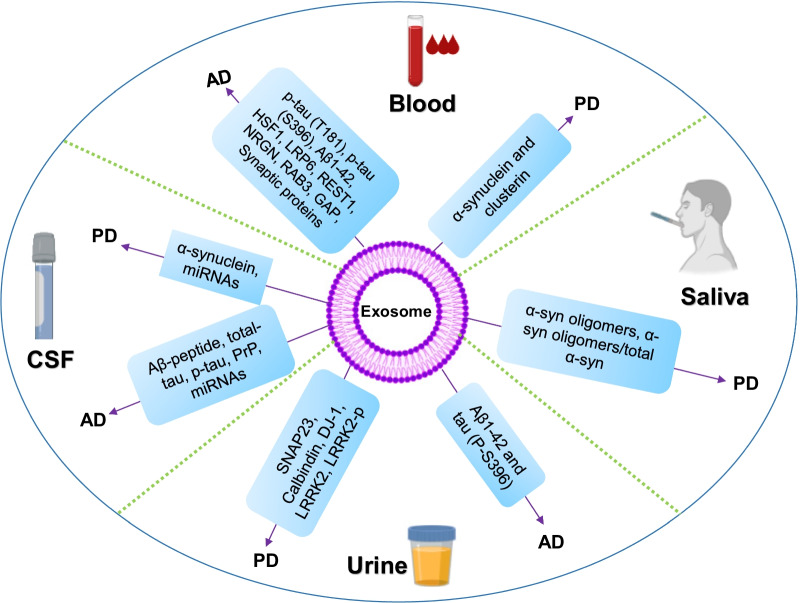
Overview of fluid-based exosomal biomarkers examined in AD and PD (created in Biorender.com)

More research efforts are required to identify the source of different EVs in urine that eventually contribute to these changes in disease-linked proteins in the overall pool of EVs. Further investigations of neuron-derived urinary exosomes may provide more meaningful and sensitive biomarkers for neurodegenerative diseases. An overview of the biological fluid-based exosomes is described in Fig. 2.

## Methods for isolation of exosomes

**Fig. 3:**
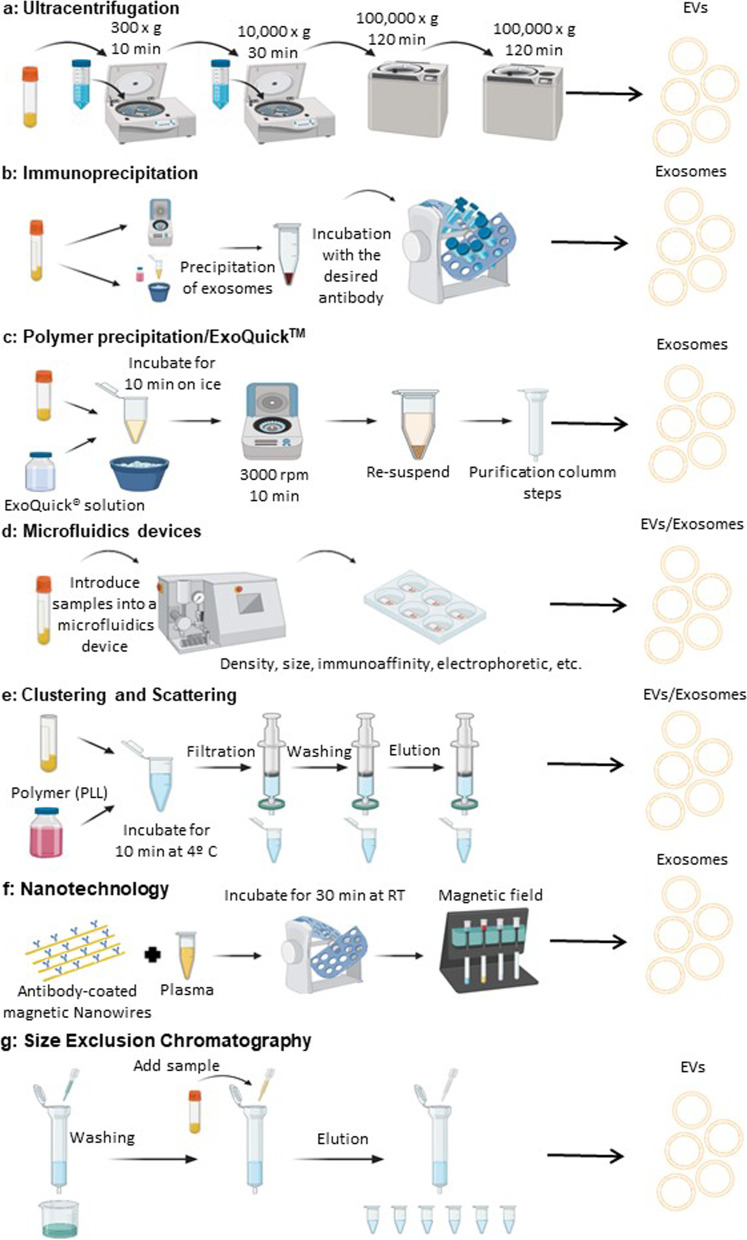
Different approaches for exosome isolation. **a** Ultracentrifugation; **b** immunoprecipitation; **c** polymer precipitation; **d** microfluidics devices; **e** clustering and scattering; **f** nanotechnology; **g** size exclusion chromatography (created in Biorender.com)

Several research efforts have been made to achieve new diagnostic tools that could be employed by clinical laboratories around the world. Quality of results, simplicity and low cost are important features to consider. Most of the current exosome isolation methods require particular and expensive equipments, are labor-intensive and time-consuming (*vide infra*), which leads to a new challenge: standardization of a protocol for clinical application in diagnostic laboratories worldwide. Currently, several techniques exist for the isolation of exosomes from human samples (Fig. 3). Here, we discuss the traditional and emerging exosome isolation techniques, and compare their advantages and disadvantages, particularly in the context of biofluid-based exosome enrichment for clinical applications.

### Ultracentrifugation

Ultracentrifugation is considered a gold-standard method and is a most widely used method for extraction of EVs [100]. Samples of body fluids or cell culture media are successively spun with increasing speeds to remove apoptotic bodies, dead cells, debris and shedding vesicles [101]. A final high-speed spinning (> 100,000 g) is mandatory to precipitate small EVs enriched mainly with exosomes due to their low density [102]. The main disadvantage of this method is the long period required to isolate exosomes. Another disadvantage is the contamination due to co-sedimentation of protein aggregates and/or nucleosomal fragments [100]. Furthermore, the low yield of the final extractions due to the repetitive ultracentrifugation steps is an extra concern [103]. Therefore, this method is not suitable for the isolation of exosomes from a small amount of blood samples [104]. To get a comparatively pure population of exosomes, ultracentrifugation has been combined with some additional steps, such as density gradient of sucrose [105] and microfiltration [106]. Addition of new steps has improved the quality of the isolated exosomes; nonetheless, the out-put has not been satisfactory yet. Given the low yield of brain cell-specific exosomes (e.g. neuronal exosomes ≤ 1% of the total blood exosomes, in the blood) [12], ultracentrifugation is less applicable.

### Immunoaffinity-based approaches

Exosomes contain several transmembrane proteins, such as CD9, CD171, CD81, CD82, RAB5, annexin, and epithelial cell adhesion molecule, which can be used as specific markers for the extraction of specific exosome populations [46, 107]. Immunoprecipitation protocol can be used to purify cell-specific exosomes (i.e., brain-derived exosomes from blood samples) by targeting surface markers. Recently, Zhang’s [108] and Goetzl’s [70] groups developed immunochemical methods to collect neuronal exosomes from small volumes of plasma using L1CAM (CD171) and NCAM as a surface capture marker [109].

Immunoprecipitation has also been used to extract EVs from other specimens, including tumor [110] and placental EVs [111]. Although immunoprecipitation can be used to isolate exosomes with comparatively higher purity, selection of the surface marker is quite challenging given the fact that surface markers are present in multiple post-translationally modified forms (e.g. cleavage products) in the body fluids [73] and are not only restricted to the brain cells. As CNS-derived exosomes represent a small fraction of the total exosomes present in the blood, ultra-sensitive detection is a prerequisite for the establishment of exosome-based diagnostics [12].

### Polymer precipitation

Commercial kits used for the precipitation of exosomes have been widely expanded as a one-step routine protocol for researchers. Several companies are working on developing fast and easy methods to isolate EVs from biofluids and one such example is ExoQuick (System Biosciences, USA). This kit is used to precipitate exosomes into a pellet using a combination of polymers, which are later submitted to a “purification column” in order to reduce contaminants such as immunoglobulin G (IgG) and albumin [102]. This method is simple and fast and requires only basic lab equipment [112]. The disadvantages of this method are low purity and formation of large aggregates [107, 113]. Also, ExoQuick kit is expensive, and only allows the analysis of a small number of samples, putting a substantial financial burden on clinics [104].

### Microfluidic devices

Microfluidics is the manipulation of fluid flow at micro-scales [100]. Several types of microfluidic-based devices have been designed to improve the efficiency of exosome isolation methods [114]. Various sorting mechanisms can be employed in these devices based on both the physical and biochemical properties of exosomes. In addition to traditional approaches e.g. size-, density- and immunoaffinity, advanced sorting mechanisms such as electrophoretic, electromagnetic [115] and acoustic [116] manipulations can be applied.

With the advancement in microfluidics technology, significant reductions in sample volume, isolation time and reagent consumption have been reported [117]. One such example is the DeMEA platform developed by Jung’s group. It is based on detachable microfluidic device implemented with electrochemical aptasensor, which allows rapid processing (< 1 h) using low amounts of sample (10 µl) [118]. Despite their great advancements, current microfluidic devices have some limitations in scalability, standardization and validation. Additionally, sample pretreatments, low yield or low specificity may impede downstream analysis [114].

### Clustering and scattering

Shin’s [119] group developed a new technique, which is a cost-effective and simple method used to isolate exosomes from blood samples. It is based on the imitation of liquid chromatography methodology. The principle behind this methodology relies on a combination of size-, charge-, and chaotic-based mechanisms that allow the isolation of EVs from biological fluids. It involves adding a cationic polymer to the sample, so that negatively charged EVs aggregate with the polymer. After a 10-min incubation period, the sample goes through a filtration step, in which aggregated EVs remain on the syringe filter, while small fragments and proteins are flushed out. To recover EVs, a chaotropic agent (guanidium thiocyanate) is used as an elution buffer. The polymers are captured on an anionic membrane, allowing the isolation of purified and concentrated EVs.

The authors describe that this method has several advantages compared to the conventional methods including ultracentrifugation, charge-interaction and polymer-precipitation methods. The main advantage is its high scalability; it can handle a range of sample volumes from 10 μl to 50 l. Furthermore, it provides a high yield (20-fold) and purity ratio (3.5) compared to ultracentrifugation. In comparison to commercially available kits, this method offers 2- to 20-folds higher performance, and size-selected isolation of EVs is possible. However, this method cannot be used for cell-specific isolation of exosomes, which is beneficial for the diagnosis of CNS diseases.

### Nanotechnology

In addition to the above methods, the application of the nanotechnology toolkit not only provides new opportunities for better diagnostic strategies, but also provides new insights into the pathophysiology of EVs [120, 121]. The recent advancements in nanotechnology, especially the utilization of multifunctional nanostructures to isolate exosomes from body fluids of cancer patients [122, 123], shed light on the contribution of nanotechnology in the development of ultrasensitive and efficient methods.

Recently, many new isolation methods based on nanotechnology have been reported. A recent study has reported a novel and ultrasensitive method, which increased the capture efficiency of cell-specific exosomes to approximately three folds compared to conventional methods. In this method, the application of antibody-coated magnetic nano-wires gives the flexibility to conjugate diverse exosome-specific antibodies, facilitating cell-specific exosome isolation [124]. Furthermore, a combination of membrane-based exosome separation with streptavidin-modified iron oxide nano-particles (SA-IONPs) has been used for rapid and efficient isolation of MVs [125]. Kabe and his colleagues described a new methodology called ExoCounter, which can count exosomes derived from a human cell or sera* via* nanobead-labeled exosomes on an optical disc [126]. More recently, Nemati et al. used magnetic nanoplatforms (magnetic nanowires, Nano rods and magnetosomes) to isolate tumor-derived exosomes [121]. They reported a greater efficiency of magnetic nanowires in comparison to rods and magnetosomes.

### Size exclusion chromatography (SEC)

The SEC technique is becoming a method of choice to obtain high-quality exosomes [127]. This technique separates vesicles according to their size. It uses starting biofluid as a mobile phase and a porous gel filtration polymer as a stationary phase [128]. A differential elution is accomplished by the stationary phase, where bigger particles are eluted first, followed by smaller ones and non-membrane-bound proteins in the end.

This is a single-step isolation system [128] with a very short processing time (~ 20 min per sample) [129]. SEC is very efficient for the isolation of relatively pure EVs, mainly by reducing contaminating plasma protein and high-density lipoproteins [130]. Another advantage of SEC is that it leads to the superior integrity of isolated exosomes as it uses gravity rather than sheer force as an isolation method [131]. One of the main disadvantages of this method is that it cannot differentiate between exosomes and MVs of comparable size. For isolation of subtype-specific exosomes, a combination of immunocapture methods is required. To isolate substantial amounts of subtype-specific exosomes, large amounts of starting materials are required [132].

These methods are great efforts in addition to classical magnetic bead capture, to significantly improve the isolation efficiency of EVs including exosomes. A combination of methods is required to isolate specific exosomes (not EVs). Multiple research centers and laboratories across the world are using all the above-described approaches. However, standardized methods that can be used in diagnostic laboratories as regular techniques are still lacking. Table 1 shows the side-by-side comparison of the currently available methods and relative extent of their development, e.g., purity, time, scalability, throughput and cost; all aspects are important to bring exosome technology into clinics.

**Table 1:** Comparison of exosome isolation methods for clinical applications

Method	Yield/purity	Time	Consumables/equipment	Advantages	Disadvantages
Ultracentrifugation	Low	2–3 h	Low cost for reagents	Classical method	Large sample volumes are required
			Requires an ultracentrifuge	Standardized protocol	Time-consuming
				Large sample capacity	Further purification steps are required
					Only well-equipped laboratories can use
Immunoaffinity-based approaches	Low yield, high purity	4–12 h	High cost for reagents	Allow enrichment of cell-specific exosomes by targeting surface markers	Time-consuming
			Commercial kits are available	Require low sample volume	Selection of markers is challenging
			No special lab equipment is required	Commercial kits are available	
Polymer precipitation	High	4–24 h	High cost for reagents	Commercial kits are available	Time-consuming
			Commercial kits are available	Requires low sample volume	Expensive
			No special equipment is required		
Microfluidic devices	High	Depends on the technology	Very high cost for development of technology	Various sorting mechanisms can be employed	Require trained and skilled personnel
			No commercial kits are available		Need standardization and validation
			Must be designed by researchers themselves		Scalability is a problem currently
Clustering and scattering	High	20 min	Low cost for reagents	Allows a wide range of sample volume	Need validation
			No special equipment is required	High scalability and purity	
Nanotechnology (nanowires)	High	30 min	High cost for reagents	High purity	Does not allow a large amount of sample
			Commercially available nanowires	Small sample volumes are required	
Size exclusion chromatography	Low yield, high purity	20 min	High cost for reagents	High purity	Large sample volumes are required for subtyping
			Commercial columns are available	High integrity	Low yield
					Expensive

## Conclusion

Exosome research is a rapidly developing field. Over the past decades, exosomes have emerged from the initial characterization as the “trash bins” of cells into key players in many pathophysiological processes. Exosomes are particularly appealing for the diagnosis of neurodegenerative diseases because they can cross the blood–brain barrier and harbor markers that can be used to track their cells of origin. A great progress has been made in this direction, particularly for AD and PD. Although prion protein was detected in exosomes quite long ago, research efforts towards the utility of exosomes as a source of biomarkers for prion diseases are still lacking. Detection of pathological proteins not only in blood-derived brain exosomes, but also in urine and salivary exosomes at early stages suggests a great potential of exosomes to act as early non-invasive biomarkers for neurodegenerative diseases. Using peripheral biological fluids as a source of CNS-derived exosomes for diagnosis, prognosis and progression of a disease is advantageous. However, there are caveats in the development of CNS-exosome-based biomarkers for neurodegenerative diseases.

1. The amount of brain-originated exosomes is very low in peripheral fluids (e.g. neuronal exosomes ≤ 1% of the total blood-isolated exosomes).
2. There are many concerns about the "black box" nature of cell-specific exosome isolation. The question is: can a change in the cell surface proteins used to isolate cell-type exosomes affect the measured concentrations, particularly if this change is as a result of a biological process? Little is known about how exosome production is influenced by different disease states and stages. Is the observed differential expression of exosomal proteins due to an increase in the number of exosomes or the concentration of a specific protein? Further advancements in exosome pathobiology, isolation and detection methods will help to answer this question and advance the field of exosome-based diagnostics.
3. Research on brain-derived cell-specific exosomes still requires a great deal of technical advancement. The lack of an efficient, ultrasensitive and standardized purification and downstream RNA and protein analysis method is a major challenge for bringing CNS-exosome technology into clinics.

With the rapid advancements in nanotechnology tools, ultrasensitive isolation of cell-specific exosomes can provide a new paradigm for the diagnosis and treatment of neurodegenerative diseases, as has been applied in the cancer field. Although the utilization of exosomes for diagnostics in neurodegenerative diseases is still in the early stages of development, it is expected that through further research efforts focusing on the development of ultra-efficient methods for isolation and purification, the true potential of CNS exosomes will be applied for more effective clinical disease diagnosis.

**Fig. 4:**
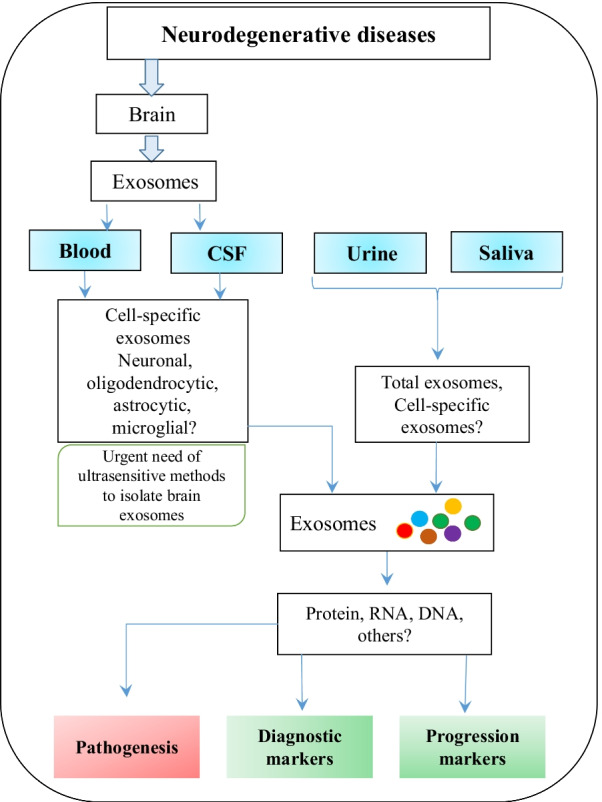
Potential outcomes of exosome-cargo investigations for neurodegenerative diseases. Identification and characterization of whole cargoes of bio-fluid-based exosomes (e.g., protein, RNA and DNA) isolated at different stages of the disease could not only provide potential novel diagnostic and prognostic markers, but also provide insights into the pathogenesis of these incurable neurodegenerative diseases

Further developments in exosome biology in combination with nanotechnology tools would offer extraordinary opportunities to overcome the current difficulties for further development of exosome-based diagnostic and therapeutic tools. An efficient and ultrasensitive detection method for exosomes will not only help discover new biomarkers, but also help understand the pathogenesis of neurodegenerative diseases at preclinical stages (Fig. 4).

## Data Availability

Not applicable.

## Abbreviations

BBB: Blood–brain barrier
DeMEA: Detachable microfluidic device implemented with electrochemical aptasensor
EVs: Extracellular vesicles
MVBs: Multivesicular bodies
